# Genomic Characterization of the Emerging Pathogen Streptococcus pseudopneumoniae

**DOI:** 10.1128/mBio.01286-19

**Published:** 2019-06-25

**Authors:** Geneviève Garriss, Priyanka Nannapaneni, Alexandra S. Simões, Sarah Browall, Karthik Subramanian, Raquel Sá-Leão, Herman Goossens, Herminia de Lencastre, Birgitta Henriques-Normark

**Affiliations:** a Department of Microbiology, Tumor and Cell Biology, Karolinska Institutet, Stockholm, Sweden; b Laboratory of Molecular Genetics, Instituto de Tecnologia Química e Biológica Antonio Xavier, Universidade Nova de Lisboa, Oeiras, Portugal; c Laboratory of Medical Microbiology, Vaccine & Infectious Disease Institute (VAXINFECTIO), University of Antwerp, Antwerp, Belgium; d Laboratory of Microbiology and Infectious Diseases, The Rockefeller University, New York, New York, USA; e Lee Kong Chian School of Medicine (LKC) and Singapore Centre on Environmental Life Sciences Engineering (SCELSE), Nanyang Technological University, Singapore; f Clinical Microbiology, Karolinska University Hospital, Bioclinicum, Stockholm, Sweden; University of Würzburg; Emory University; University of Greifswald

**Keywords:** infectious disease, *Streptococcus pseudopneumoniae*, *Streptococcus pneumoniae*, bacterial diagnostics, comparative genomics

## Abstract

Streptococcus pseudopneumoniae is a close relative of the major human pathogen S. pneumoniae. It is increasingly associated with lower-respiratory-tract infections (LRTI) and a high prevalence of antimicrobial resistance (AMR). *S. pseudopneumoniae* is difficult to identify using traditional typing methods due to similarities with S. pneumoniae and other members of the mitis group (SMG). Using whole-genome sequencing of LRTI isolates and a comparative genomic approach, we found that a large number of pneumococcal virulence and colonization genes are present in the core *S. pseudopneumoniae* genome. We also reveal an impressive number of novel surface-exposed proteins encoded by the genome of this species. In addition, we propose a new and entirely specific molecular marker useful for the identification of *S. pseudopneumoniae*. Phylogenetic analyses of *S. pseudopneumoniae* show that specific clades are associated with allelic variants of core proteins. Resistance to tetracycline and macrolides, the two most common types of resistance, were found to be encoded by Tn*916*-like integrating conjugative elements and Mega-2. Overall, we found a tight association of genotypic determinants of AMR and phenotypic AMR with a specific lineage of *S. pseudopneumoniae*. Taken together, our results shed light on the distribution in *S. pseudopneumoniae* of genes known to be important during invasive disease and colonization and provide insight into features that could contribute to virulence, colonization, and adaptation.

## Abstract (precis)

*S. pseudopneumoniae* is an overlooked pathogen emerging as the causative agent of lower-respiratory-tract infections and associated with chronic obstructive pulmonary disease (COPD) and exacerbation of COPD. However, much remains unknown on its clinical importance and epidemiology, mainly due to the lack of specific markers to distinguish it from S. pneumoniae. Here, we provide a new molecular marker entirely specific for *S. pseudopneumoniae* and offer a comprehensive view of the virulence and colonization genes found in this species. Finally, our results pave the way for further studies aiming at understanding the pathogenesis and epidemiology of *S. pseudopneumoniae*.

## INTRODUCTION

Streptococcus pseudopneumoniae is a close relative of the human pathogen S. pneumoniae, and it was first described in 2004 (1). It belongs to the mitis group along with 13 other species, including some of the most common colonizers of the oral cavity, such as S. mitis (2). An increasing number of reports indicate that *S. pseudopneumoniae* is a potential pathogen, usually associated with underlying medical conditions (3–5). It can be isolated from multiple invasive and noninvasive sites (6–9) and was reported as the probable causative agent in fatal septicemia cases (5). Experiments using multiple *S. pseudopneumoniae* strains in a mouse peritonitis/sepsis model have further underlined its pathogenic potential (10). *S. pseudopneumoniae* is also frequently associated with high rates of antimicrobial resistance (AMR), in particular to penicillin, macrolides, co-trimoxazole, and tetracycline (6–8).

Despite its emerging role as a pathogen, relatively little is known about the epidemiology, pathogenic potential, and genetic features of *S. pseudopneumoniae*. This problem is partially attributable to difficulties in distinguishing it from S. pneumoniae and S. mitis, highlighted by the incorrect identification of 50% of the publicly available genome sequences of *S. pseudopneumoniae* (11, 12). It is likely that infections due to *S. pseudopneumoniae* are overlooked or misdiagnosed due to lack of reliable measures to identify this species. *S. pseudopneumoniae* was originally described as optochin resistant if grown in the presence of 5% CO_2_ but susceptible in ambient atmosphere, bile insoluble, and nonencapsulated (1), but exceptions to these phenotypes were later reported (4, 5, 7, 13). Molecular methods, such as PCR amplification of specific markers, mostly aim at identifying pneumococci and, thus, have limited value for the positive identification of *S. pseudopneumoniae*. The only molecular marker reported so far for the identification of *S. pseudopneumoniae*, SPS0002, is also found in a subset of S. pneumoniae strains (12). Understanding the clinical significance and epidemiology of *S. pseudopneumoniae* requires more discriminative identification methods and a more complete picture of its genetic diversity.

All *S. pseudopneumoniae* strains described to date lack a polysaccharide capsule, which is considered the major virulence factor of S. pneumoniae due to its inhibitory effect on complement-mediated opsonophagocytosis. In addition to the capsule, a plethora of other factors, and especially surface-exposed proteins, have been shown to significantly contribute to pneumococcal disease and colonization (reviewed in references 14 and 15), and some of these features have been identified in *S. pseudopneumoniae* (3, 9, 14, 16). Despite the lack of a capsule, naturally nonencapsulated pneumococci can cause disease, and the surface protein PspK, expressed by a subgroup of nonencapsulated pneumococci, promotes adherence to epithelial cells and mouse nasopharyngeal colonization to levels comparable with those of encapsulated pneumococci (17, 18). A comprehensive overview of the distribution of known and potentially new genes that could promote virulence and colonization in *S. pseudopneumoniae* is, however, still lacking.

In this study, we performed an extensive comparative genomic analysis with the aim of elucidating the molecular features that characterize *S. pseudopneumoniae* and distinguish it from its close relative, S. pneumoniae. We show that a substantial number of known pneumococcal virulence factors are conserved in *S. pseudopneumoniae*, and we identify a vast number of novel surface-exposed proteins. Finally, our results establish a tight association of AMR determinants with certain lineages and reveal the composite scenario of genetic elements that characterize this species. Importantly, we identified a genetic marker uniquely present in *S. pseudopneumoniae* that can allow the identification of this overlooked species.

## RESULTS

### Identification of *S. pseudopneumoniae* genomes.

**FIG 1:**
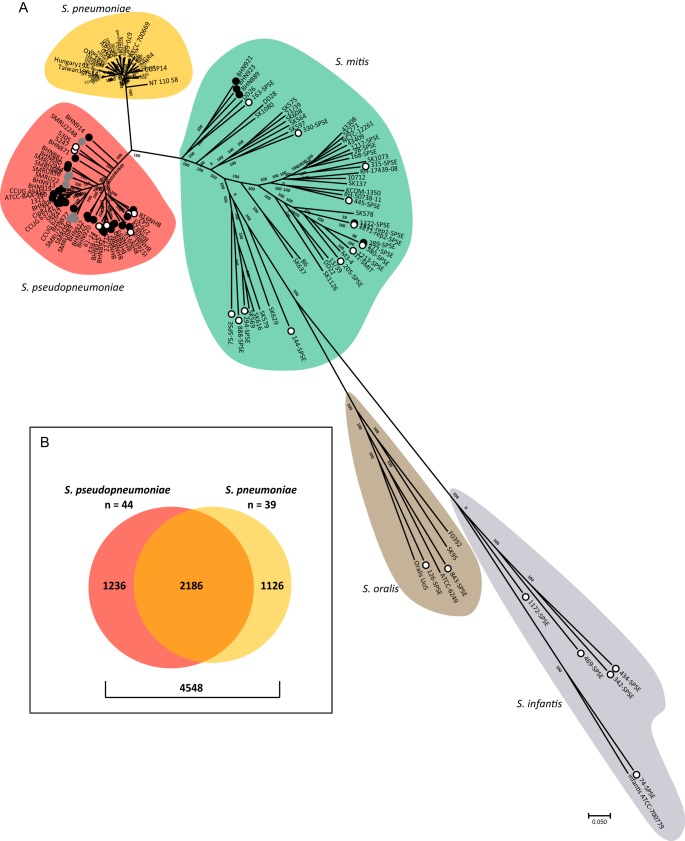
Phylogenetic and pangenome analysis of *S. pseudopneumoniae*. (A) Unrooted consensus parsimony phylogenetic tree based on all SNPs (1,230,968) of 147 genomes: LRTI isolates (*n* = 24) and publicly available *S. pseudopneumoniae* (*n* = 38), S. pneumoniae (*n* = 39), nontypeable S. pneumoniae (*n* = 8), S. mitis (*n* = 36), S. oralis (*n* = 1), and S. infantis (*n* = 1). Circles indicate LRTI isolates (black), NCBI genomes labeled as *S. pseudopneumoniae* (open), or nontypeable S. pneumoniae (gray). Background shading delineates clades of different species. The tree was built in kSNP and visualized in MEGA7 (51). (B) Pangenome of *S. pseudopneumoniae* and S. pneumoniae showing the distribution of shared and unique COGs.

Whole-genome sequencing (WGS) followed by a phylogenetic analysis, including 147 genomes from various streptococci of the mitis group (SMG) species, was performed to classify 24 isolates collected from lower-respiratory-tract infections (LRTI) (19) that we suspected to be *S. pseudopneumoniae* (*n* = 16) or S. mitis (*n* = 3) or for which no definitive classification was possible to obtain using traditional typing methods and multilocus sequence analysis (MLSA) (*n* = 5). Twenty-one of 24 LRTI isolates clustered within the *S. pseudopneumoniae* clade (Fig. 1A). The 3 strains initially identified as S. mitis clustered within the S. mitis clade and are not discussed further in this study. As previously reported (11, 12), 8 nontypeable S. pneumoniae NCBI genomes fell within the *S. pseudopneumoniae* clade, along with only 15/38 publicly available genomes currently classified as *S. pseudopneumoniae* (Fig. 1A). Based on our phylogenetic analysis, a total of 44 sequenced genomes were considered *S. pseudopneumoniae* and further analyzed (see Data Set S1 in the supplemental material). The pangenome of these 44 *S. pseudopneumoniae* genomes is composed of 3,447 clusters of orthologous genes (COGs), of which 44% are found in the core genome (≥95% isolates) and 56% in the accessory genome.

10.1128/mBio.01286-19.1 DATA SET S1 List of *S. pseudopneumoniae* genomes included in this study. Download Data Set S1, XLSX file, 0.02 MB. Copyright © 2019 Garriss et al. 2019 Garriss et al. This content is distributed under the terms of the Creative Commons Attribution 4.0 International license.

### A single locus, SPPN_RS10375, can be used to identify *S. pseudopneumoniae*.

We then aimed to identify COGs present in *S. pseudopneumoniae* but absent from S. pneumoniae. We first defined the pangenome of these two species, using the 44 *S. pseudopneumoniae* genomes and 39 completed and fully annotated S. pneumoniae NCBI genomes, and found that 2,186/4,548 COGs (48%) were shared by both species (Fig. 1B). We identified 30 core COGs, present in each of the 44 *S. pseudopneumoniae* genomes, among the 1,236 COGs unique to *S. pseudopneumoniae* (Table S1). We then assessed the presence of each of these 30 COGs in other bacterial species by BLASTn analysis against all NCBI genomes, including the 8,358 S. pneumoniae genomes deposited at the time of the study. This revealed that only two COGs, represented by open reading frames (ORFs) SPPN_RS10375 and SPPN_RS06420, were found exclusively in *S. pseudopneumoniae* genomes. While SPPN_RS06420 has a G+C content challenging for the design of PCR primers (average of 27.1%), further analysis of SPPN_RS10375, which encodes a hypothetical protein, and its surrounding intergenic regions in the 44 genomes indicated that this 627-bp locus is a suitable candidate for a molecular marker. Eight clinical isolates not subjected to whole-genome sequencing and collected during the same LRTI study (19) that were either impossible to identify (*n* = 4) or suspected to be *S. pseudopneumoniae* (*n* = 4) were found to be positive by PCR for SPPN_RS10375, indicating that they belong to the *S. pseudopneumoniae* species (Fig. S1A). These strains were also positive for the recently published *S. pseudopneumoniae* marker SPS0002 (12) (Fig. S1B).

10.1128/mBio.01286-19.3 TABLE S1 *S. pseudopneumoniae* molecular marker candidates. Download Table S1, PDF file, 0.04 MB. Copyright © 2019 Garriss et al. 2019 Garriss et al. This content is distributed under the terms of the Creative Commons Attribution 4.0 International license.

10.1128/mBio.01286-19.6 FIG S1 Molecular typing of *S. pseudopneumoniae.* (A and B) Agarose gels showing PCR products from the amplification of SPPN_RS10375 (402 bp) (A) and the previously published marker SPS0002 (119 bp) (M. A. Croxen, T. D. Lee, R. Azana, and L. M. Hoang, Microb Genom 4:e000175, 2018, https://doi.org/10.1099/mgen.0.000175) (B) in a control panel of *S. pseudopneumoniae* (BHN868 and BHN880), S. pneumoniae (TIGR4, R6, and BHN46), S. mitis (BHN889), nontypeable S. pneumoniae (BHN1341, BHN1342, and BHN1343), suspected *S. pseudopneumoniae* isolates (BHN1333 to BHN1336), and isolates not typeable by MLSA (BHN1337 to BHN1340). M, molecular weight marker. (C) Analysis of regions targeted by PCR and RFLP for discriminating between pneumococcal and atypical *lytA* genes. S. pneumoniae TIGR4 binding sites of primers lytA-A750 and lytA-A1145 (D. Llull, R. Lopez, and E. Garcia, J Clin Microbiol 44:1250–1256, 2006, https://doi.org/10.1128/JCM.44.4.1250-1256.2006) used for PCR amplification of pneumococcal *lytA* were aligned with chromosomal and phage-located *lytA* genes from strain BHN892. Red letters indicate nucleotide mismatches, and nucleotide positions in TIGR4 *lytA* are indicated above the sequence. The total numbers of mismatches in the primer binding sites are indicated, as well as the overall nucleotide identity of each *lytA* gene with the TIGR4 *lytA* strain. The number of nucleotides separating the two primer binding sites is indicated in each sequence. Underlined nucleotides designate the BsaAI restriction site used for RFLP analysis, and the triangle indicates the cleavage site. Download FIG S1, PDF file, 0.9 MB. Copyright © 2019 Garriss et al. 2019 Garriss et al. This content is distributed under the terms of the Creative Commons Attribution 4.0 International license.

Among the 29 *S. pseudopneumoniae* LRTI isolates, 16 (55%) displayed the typical optochin susceptibility and bile solubility phenotypes originally attributed to this species (1) (Table 1). As previously described (7), the pneumococcus-specific markers 16S rRNA and *spn9802* were positive in the majority of the isolates. Discrepancies between the restriction fragment length polymorphism (RFLP) and PCR results used for detecting the pneumococcal variant of the autolysin gene *lytA* were found to be due to phage-encoded *lytA* genes similar enough to be identified by PCR as the pneumococcal *lytA* but lacking the BsaAI restriction site used for RFLP analysis (20) (Fig. S1C). The pneumococcal variant of the cytotoxin pneumolysin gene *ply* was detected by RFLP in three instances. However, this is due to the presence of the BsaAI restriction used for RFLP in some nonpneumococcal variants of Ply (see below and Fig. S2).

**TABLE 1:** Phenotypic and genotypic characterization of *S. pseudopneumoniae* LRTI isolates

Parameter	No. (%) of strains (*n* = 29)
Phenotypic markers	
Optochin susceptibility	
5% CO_2_	3 (10.3)
Ambient atmosphere	19^*a*^ (65.5)
Bile solubility	1^*b*^ (3.4)
Genotypic markers	
PCR markers	
Pneumococcal *lytA*	2 (6.9)
*cpsA*	0 (0.00)
*spn9802*	28 (96.5)
Pneumococcus-specific 16S rRNA	25 (86.2)
RFLP signatures	
Pneumococcal/atypical *lytA*	0/29 (0.100)
*ply*-*mly*	5/24 (17.2/82.8)

a Eight strains did not grow in ambient atmosphere. The 18/19 strains susceptible in ambient atmosphere were resistant in CO_2_.

b Two strains showed partial solubility.

10.1128/mBio.01286-19.7 FIG S2 Phylogenetic tree of 93 Ply alleles from SMG strains. MEGA7 (S. Kumar, G. Stetcher, and K. Tamura, Mol Biol Evol 33:1870–1874, 2016, https://doi.org/10.1093/molbev/msw054) was used to infer the evolutionary history using the maximum likelihood method based on the JTT matrix-based model (D. T. Jones, W. R. Taylor, and J. M. Thornton, Comput Appl Biosci 8:275–282, 1992). The tree with the highest log likelihood (−1,453.23) is shown. There were a total of 245 positions in the final dataset. Leafs are colored based on the species: yellow, S. pneumoniae; red, *S. pseudopneumoniae*; green, S. mitis. Ply clades are indicated by the background shading: grey, pneumococcal Ply; blue, atypical (Mly/Pply). Asterisks indicate Ply variants outside the S. pneumoniae Ply clade that would be classified as pneumococcal Ply based on the presence of the BsaAI restriction site used for RFLP analysis (D. Rolo, A. S. Simoes, A. Domenech, A. Fenoll, et al., PLoS One 8:e57047, 2013, https://doi.org/10.1371/journal.pone.0057047, and A. S. Simoes, R. Sa-Leao, M. J. Eleveld, D. A. Tavares, et al., J Clin Microbiol 48:238–246, 2010, https://doi.org/10.1128/JCM.01313-09). Download FIG S2, EPS file, 1.4 MB. Copyright © 2019 Garriss et al. 2019 Garriss et al. This content is distributed under the terms of the Creative Commons Attribution 4.0 International license.

### Pneumococcal virulence and colonization genes are widely distributed in *S. pseudopneumoniae*.

**FIG 2:**
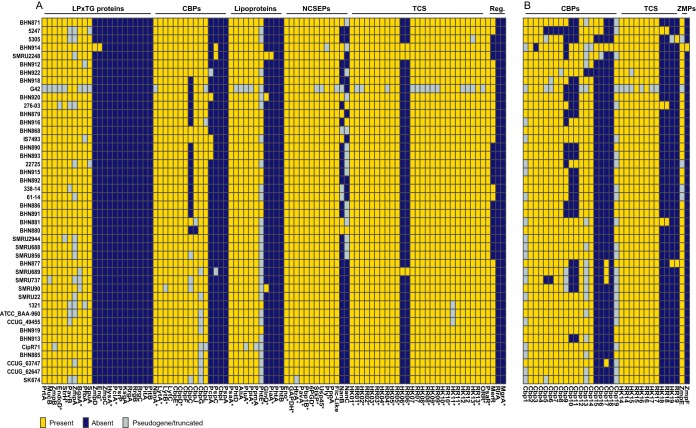
Distribution of known and putative new colonization and virulence factors in the 44 *S. pseudopneumoniae* genomes. (A) Known pneumococcal surface-exposed proteins, two-component systems (histidine kinase/response regulator pairs), and stand-alone regulators. Asterisks indicate core S. pneumoniae proteins according to Gamez et al. (21). (B) Distribution of novel choline-binding proteins, two-component systems, and zinc-metalloproteases. CBP, choline-binding protein; NCSEP, nonclassical surface-exposed protein; TCS, two-component system; HK, histidine kinase; RR, response regulator; Reg., stand-alone regulator; ZMPs, zinc metalloproteases.

**FIG 3:**
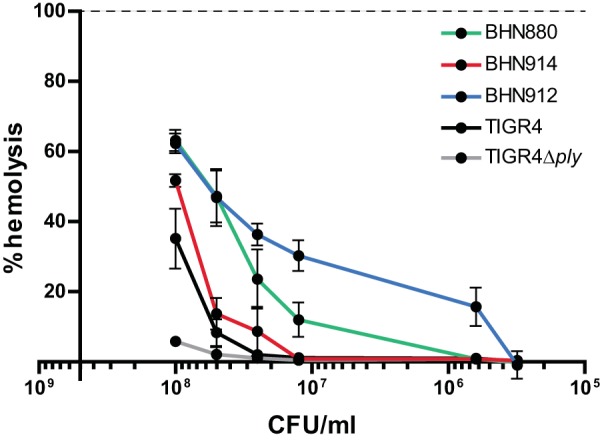
Hemolytic activity of *S. pseudopneumoniae*. The ability of *S. pseudopneumoniae* strains BHN880, BHN912, and BHN914 to lyse human blood was tested using a hemolysis assay. S. pneumoniae TIGR4 and TIGR4Δ*ply* strains were used as controls. Results are expressed as the percentage of lysis compared to the positive control (100%; indicated by the dashed line) upon incubation with decreasing numbers of CFU/ml. Each data point represents the average from three independent experiments, and standard deviations are indicated.

To gain insight into genetic features that could promote adhesion, virulence, and colonization, we investigated the presence of orthologues of 92 pneumococcal surface-exposed proteins, transcriptional regulators, and two-component signal transducing systems (TCSs) for which the distribution among pneumococcal genomes has been studied (21, 22). No orthologs were found in *S. pseudopneumoniae* for 16/92 proteins, including the subunits of both pneumococcal pili (RrgABC and PitAB), surface-exposed proteins PsrP and PspA, and the stand-alone regulators MgrA and RlrA (Fig. 2A). Three of these sixteen proteins, HysA, PclA, and MgrA, are core S. pneumoniae features (21). Other core S. pneumoniae proteins were represented in only a very small subset of *S. pseudopneumoniae* strains, such as Eng (*n* = 1), PiaA (*n* = 1), GlnQ (*n* = 3), and the histidine kinase (HK) and response regulator (RR) that constitute TCS06 (*n* = 3). Of 61 surface-exposed proteins, 29 were found in the core *S. pseudopneumoniae* genome, including major virulence factors such as pneumolysin (Ply), NanA, and HtrA (Fig. 2A). The NanA variant found in *S. pseudopneumoniae* shares similar domains and has good similarity with pneumococcal NanA (61.2%). However, it differs strongly in its C-terminal region, where the LPxTG-anchoring domain normally found in S. pneumoniae NanA is replaced with a choline-binding domain (CBD). The *S. pseudopneumoniae* Ply proteins (sometimes referred to as Pply) are extremely well conserved (99.1% pairwise identity). Interestingly, while S. pneumoniae and *S. pseudopneumoniae* carry Ply in their core genome, it is found in only a small fraction (8%) of S. mitis strains. Although they are closely related to pneumococcal Ply (97.4% pairwise identity), phylogenetic analysis shows that all *S. pseudopneumoniae* and S. mitis Ply variants fall in a phylogenetic clade distinct from that of their pneumococcal counterparts (7, 9) (Fig. S2). Hemolysis assays show that *S. pseudopneumoniae* strains encoding Ply proteins from each phylogenetic clade have a hemolytic activity comparable or superior to that of the reference S. pneumoniae TIGR4 strain (Fig. 3).

Pneumococcal LPxTG cell wall-anchored proteins were found to have the lowest levels of representation in *S. pseudopneumoniae*, with 12/23 being absent from all genomes. With the exception of TCS06 and HK11, all HK-RR pairs were core *S. pseudopneumoniae* proteins. Two of the three isolates encoding TCS06 also harbor a PspC-like protein in the same locus, such as that found in pneumococcal genomes. These two PspC-like proteins carry an LPxTG-anchoring domain and share limited similarity to each other (30.8%) and to their closest pneumococcal allele, PspC11.3 (32.9%) (23). The third genome encoding TCS06 carries a truncated gene encoding a PspC-like protein.

**FIG 4:**
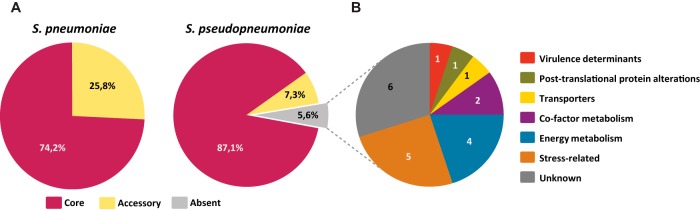
Distribution of IPD and ECC genes. (A) Pie charts representing the percentage of core, accessory, and absent genes from S. pneumoniae (*n* = 39) and *S. pseudopneumoniae* (*n* = 44) genomes. Due to the use of draft *S. pseudopneumoniae* genomes compared with complete S. pneumoniae genomes, genes were considered core in *S. pseudopneumoniae* when they were found in ≥95% of the genomes. (B) Distribution of the 20 genes absent from *S. pseudopneumoniae* in the functional categories defined by Orihuela et al. (24).

We further evaluated the presence in *S. pseudopneumoniae* of genes relevant for infection and colonization by investigating the presence of 356 S. pneumoniae genes differentially expressed in mouse models of invasive disease (IPD) and during epithelial cell contact (ECC) (24). We found that 94% are present in at least one *S. pseudopneumoniae* genome (Data Set S2). The use of draft *S. pseudopneumoniae* genomes (43/44), in contrast to fully assembled S. pneumoniae genomes, would likely result in an underestimation of the presence of these genes due to contig breaks. Hence, we considered genes present in 42/44 *S. pseudopneumoniae* genomes to belong to the core genome. A large fraction of IPD/ECC genes were found in the core genome of *S. pseudopneumoniae* (87%) and S. pneumoniae (74%) (Fig. 4A and Table S3). Of the 356 genes, 20 (5.6%) were absent from *S. pseudopneumoniae*, and among them was the gene encoding pneumococcal surface protein A (*pspA*), a known virulence factor present in the majority of S. pneumoniae genomes (34/39). The remaining genes belonged to various functional categories (Fig. 4B and Data Set S2).

10.1128/mBio.01286-19.2 DATA SET S2 Distribution of 356 genes differentially expressed in invasive pneumococcal disease (IPD) and epithelial cell contact (ECC) in *S. pseudopneumoniae* and S. pneumoniae completed genomes. Download Data Set S2, XLSX file, 0.03 MB. Copyright © 2019 Garriss et al. 2019 Garriss et al. This content is distributed under the terms of the Creative Commons Attribution 4.0 International license.

10.1128/mBio.01286-19.5 TABLE S3 Locus tags of surface-exposed proteins, two-component systems, stand-alone regulators, and zinc metalloproteases. Download Table S3, PDF file, 0.04 MB. Copyright © 2019 Garriss et al. 2019 Garriss et al. This content is distributed under the terms of the Creative Commons Attribution 4.0 International license.

### Identification of an encapsulated *S. pseudopneumoniae* strain.

**FIG 5:**
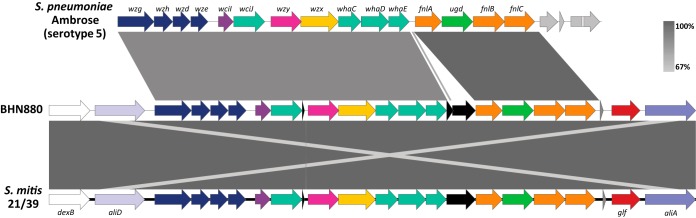
Pairwise alignment of the capsule locus of *S. pseudopneumoniae* strain BHN880 with S. pneumoniae Ambrose (serotype 5) and S. mitis 21/39. Colors and annotations are based on Bentley et al. (54). Gray shading indicates the degree of pairwise nucleotide identity. Locus tags of *dexB* and *aliA* homologs in BHN880 are E3V35_07635 and E3V35_07740, respectively.

Unexpectedly, our analysis revealed the presence of the capsular polysaccharide biosynthesis genes *cpsA* and *cpsC* in one instance (Data Set S2). Further analysis showed that one LRTI *S. pseudopneumoniae* isolate, BHN880, encodes a full capsular locus similar to the pneumococcal serotype 5 capsule and to the capsule locus of S. mitis strain 21/39 (Fig. 5). Gel diffusion assays typed BHN880 as pneumococcal serotype 5, which is supported by the high nucleotide identity (97.7%) between the regions encoding the sugar precursors of BHN880 and serotype 5 capsular loci. The 43 remaining genomes do not encode a capsule and carry the NCC3-type locus, which encompasses genes *dexB*, *aliD*, and *glf* (also known as *cap* or *capN* [18, 25]).

### *S. pseudopneumoniae* encodes a substantial number of new potential surface-exposed proteins and two-component systems.

**FIG 6:**
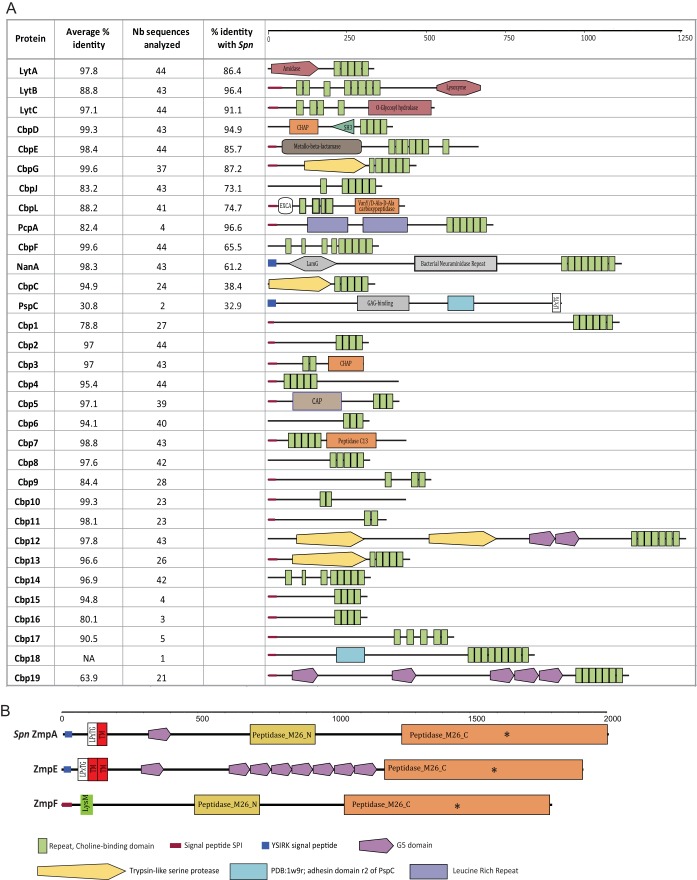
Domain prediction of choline-binding proteins and new zinc metalloproteases of *S. pseudopneumoniae* based on SMART (57). (A) Choline-binding proteins. The average percent identity of each CBP in *S. pseudopneumoniae* and the number of proteins analyzed are indicated. Percent identity with S. pneumoniae (*Spn*) was calculated using the proteins from IS7493 and S. pneumoniae TIGR4, except in the following cases: NanA (R6) and PspC (allele PspC11.3; AF276622.1). Representations of domains found in each CBP are based on the variant found in IS7493. In the absence of the protein in IS7493, the analysis was based on BHN914 (PspC, Cbp15, Cbp16, Cbp17, Cbp18, and Cbp19), BHN879 (Cbp1), and BHN886 (Cbp19). Nb, number. (B) Zinc metalloproteases ZmpE and ZmpF from *S. pseudopneumoniae*. ZmpA from S. pneumoniae (*Spn* ZmpA) is included for comparison. Asterisks indicate the ZMP motif HEMTH….E (26). Domain prediction is based on ZmpE from IS7493 and ZmpF from BHN914. Locus tags can be found in Table S3.

We then investigated if *S. pseudopneumoniae* harbored additional features that could be relevant for colonization and adaptation by searching the proteome of the *S. pseudopneumoniae* species for novel choline-binding proteins (CBPs) and new TCSs. We found 19 previously undescribed proteins containing a choline-binding domain (CBD), which we named Cbp1 to Cbp19. In total, 4 of the 19 proteins belong to the core genome, while the others have various levels of presence among the 44 genomes (Fig. 2B). Each strain carried between 6 and 15 new CBPs, and some *S. pseudopneumoniae* genomes carried a total of 26 CBPs. Prediction of functional domains in these proteins indicates that the majority of these proteins have an SP1 signal peptide and a C-terminal CBD composed of 2 to 9 repeats (Fig. 6A). While no functional domain could be identified in the majority of cases, some proteins contained known domains, such as the trypsin-like serine protease domain and the G5 domain, the latter of which is frequently associated with zinc metalloproteases (ZMPs) such as the IgA protease, ZmpA. In addition, we found that *S. pseudopneumoniae* encodes two new putative ZMPs containing the HEMTH…E motif (26), which we named ZmpE and ZmpF. While ZmpE is present in most of the isolates, ZmpF is found in only one strain (BHN914) (Fig. 2B). ZmpE harbors the domains typically found in ZMPs, such as the pneumococcal ZmpA (Fig. 6B). ZmpF, however, lacks the typical LPxTG motif and transmembrane domain and instead carries a LysM domain, which is thought to bind peptidoglycan.

We found six additional HK-RR pairs in the *S. pseudopneumoniae* pangenome, 4 of which are core features (Table 2 and Fig. 2B). We named these TCS14 to TCS19. A more detailed analysis of their genetic loci revealed that TCS14 is found next to genes encoding a ComC/Blp family peptide and bacteriocins. These genes that encode ComC/Blp peptides are distinct from those encoding ComC and BlpC associated with TCS12 and TCS13, respectively, present elsewhere in the *S. pseudopneumoniae* genome. The remaining five TCSs are genetically linked to genes predicted to encode ABC transporters involved in iron, potassium, and sugar transport, thiamine biosynthesis, and bacitracin export.

**TABLE 2:** Novel two-component signaling systems of *S. pseudopneumoniae*

TCS	RR^*a*^	HK^*b*^	Species of closest homologue	Family of regulators	Associated gene category
14	SPPN_RS00570	SPPN_RS00565	S. mitis	LytTR	Bacteriocins
15	SPPN_RS11635	SPPN_RS01890	S. mitis	YesN	Ferric iron transport
16	SPPN_RS03570	SPPN_RS03565	S. pseudoporcinus, S. canis	OmpR	Potassium transport^*c*^
17	SPPN_RS07705	SPPN_RS07700	S. parasanguinis	LytTR/YesN	Thiamine biosynthesis^*d*^
18	E3V59_10390	E3V59_10385	S. mitis	YesN/AraC	Sugar transport
19	E3V34_05540	E3V34_05535	S. suis	CitB	Bacitracin export

a Locus tag of the response regulator (RR). Locus tag in IS7493 was used when present.

b Locus tag of the sensor histidine kinase (HK). Locus tag in IS7493 was used when present.

c Similar to KdpD/KdpE from E. coli (61).

d Similar to TCS02 of S. thermophilus (62).

### Bacteriophages are tightly associated with *S. pseudopneumoniae*.

Among the 44 *S. pseudopneumoniae* strains, 27 carried at least one putatively full-length prophage, and the remaining 17 strains all carried phage genes, although the presence of full-length prophages could not be confirmed. Twenty-one of the full-length prophages shared a highly related novel integrase (≥90.5% nucleotide identity), which we termed *int_Sppn1_*, and in 19 cases these prophages were found integrated between SPPN_RS05275 (encoding a putative CYTH domain protein) and SPPN_RS05395 (encoding a putative GTP pyrophosphokinase) (Table S2). The integration site of the remaining 2 full-length prophages encoding Int*_Sppn1_* could not be confirmed, as they were found alone in a contig without chromosomal flanking sequences. Int*_Sppn1_* was found in the other 23 strains. However, a full-length prophage could not be confirmed in these strains. In all strains, except for strains G42 and ATCC BAA_960, *int_Sppn1_* was associated with some phage genes. Six strains carried a second putatively full-length prophage encoding an integrase closely related to that of pneumococcal group 2a prophages, *int2a* (27). These prophages were found between SPPN_RS07570 and SPPN_RS07555, which are the homologs of the genes flanking the phage group 2a integration site in pneumococci (28). Twenty-three other strains harbored *int2a*; however, the presence of more than one phage per strain severely impaired our ability to confirm the completeness of the phages they were associated with, as phage sequences were split between various contigs.

10.1128/mBio.01286-19.4 TABLE S2 List of prophages in *S. pseudopneumoniae* genomes. Download Table S2, PDF file, 0.03 MB. Copyright © 2019 Garriss et al. 2019 Garriss et al. This content is distributed under the terms of the Creative Commons Attribution 4.0 International license.

### *S. pseudopneumoniae* clades are characterized by distinct alleles of a peptide pheromone and different patterns of antibiotic resistance.

**FIG 7:**
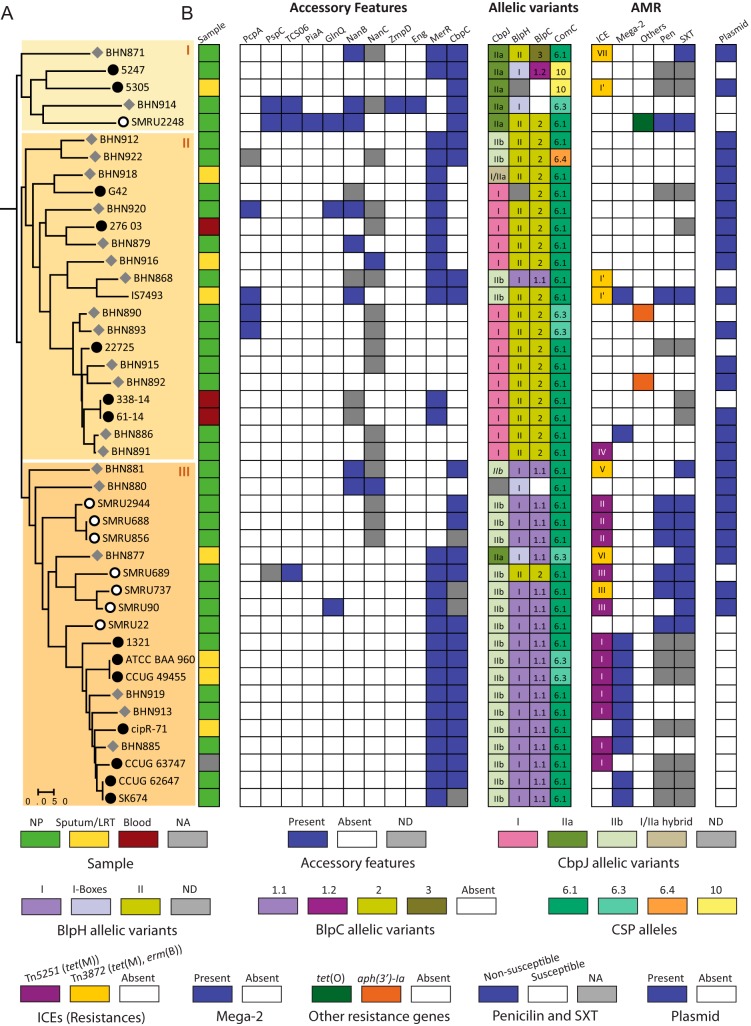
Phylogenetic distribution of accessory features and allelic variants. (A) Core genome species tree-based SNPs from the 793 single-copy core genes of 44 *S. pseudopneumoniae* genomes. Circles indicate isolates from LRTI (black), NCBI genomes labeled as *S. pseudopneumoniae* (open), or nontypeable S. pneumoniae (gray). The tree was built in panX (52) and visualized in MEGA7 (51). Clades are delineated by the background shading. (B) Distribution of accessory features and allelic variants of surface-exposed proteins, regulatory genes, peptide pheromones, genotypic and phenotypic antibiotic resistance, and plasmids. Description of the colors is indicated in the key. Supporting information on allelic variants can be found in Fig. S3B to E. Roman numerals in the ICE column refer to integration sites (Fig. S4). ICE, Mega-2, and other types of resistance refer to genotypic resistance; penicillin (Pen) and co-trimoxazole (SXT) refer to phenotypic resistance (Table 3 and references 5, 16, and 58–60). NP, nasopharynx; ND, pseudogenes/truncated; NA, data not available.

A single-nucleotide polymorphism (SNP)-based phylogenetic tree, which was constructed using the 793 *S. pseudopneumoniae* core COGs, shows that the species is divided into three clades (Fig. 7A). Clades II and III encompass most of the isolates, while clade I is composed of five isolates which fall closer to S. pneumoniae genomes (Fig. 7A and Fig. S3A). All three clades are composed of strains isolated from the nasopharynx and from sputum or lower-respiratory-tract samples. The three blood isolates belonged to clade II. No specific association between accessory virulence/colonization features and specific phylogenetic clades could be seen, except for PcpA, which was found exclusively in clade II, and CbpC, which was found in most strains of clade I and in all strains of clade III (Fig. 7B). Isolates carrying PiaA, ZmpD, and Eng, three features found only once, belonged to clade I. The presence of CbpC correlated with specific alleles of the protein encoded by the neighboring gene, CbpJ (Fig. 7B and Fig. S3B). Strains which carried variant I of CbpJ were exclusively found in clade II and in all cases were devoid of CbpC. Interestingly, the major clades II and III were characterized by distinct alleles of the histidine kinase HK13 (BlpH) and of BlpC, the peptide pheromone which controls the expression of bacteriocins in S. pneumoniae (Fig. 7B and Fig. S3C). Four variants of BlpH, which did not specifically cluster with a specific clade, were found to be similar to BlpH-I in boxes 1 and 2, which are important for interaction with BlpC (29). As expected, BlpH variants were almost strictly associated with specific variants of BlpC, BlpC*Spp*1.1 and BlpC*Spp*2. The latter is identical to BlpC 6A (29), while the former differs from BlpC R6 by one amino acid in the leader peptide sequence (Fig. S3D). Two strains carried other BlpC alleles, BlpC*Spp*1.2, which is identical to BlpC R6, and BlpC*Spp*3, which is unique. Unlike the case for BlpC, most strains had the same CSP pherotype. Besides CSP6.1 and CSP6.3, which have previously been described in *S. pseudopneumoniae* (30), 2 new alleles of ComC were found, CSP6.4 and CSP10 (Fig. 7B and Fig. S3E).

10.1128/mBio.01286-19.8 FIG S3 (A) Phylogenetic tree based on the SNPs of single-copy core genes (*n* = 610) present in the completed 39 S. pneumoniae genomes and 44 *S. pseudopneumoniae* genomes using panX (W. Ding, F. Baumdicker, and R. A. Neher, Nucleic Acids Res 46:e5, 2018, https://doi.org/10.1093/nar/gkx977). Branch coloring indicates the two species: red, *S. pseudopneumoniae*; yellow, S. pneumoniae. (B) Maximum likelihood phylogenetic tree of *S. pseudopneumoniae* CbpJ proteins. (C) Maximum likelihood phylogenetic tree of *S. pseudopneumoniae* BlpH proteins. Evolutionary analyses of CbpJ and BlpH were conducted in MEGA7 (S. Kumar, G. Stetcher, and K. Tamura, Mol Biol Evol 33:1870–1874, 2016, https://doi.org/10.1093/molbev/msw054) using multiple-sequence alignments generated in Geneious, version 10.1.3 (https://www.geneious.com), with the MUSCLE algorithm with default parameters as the input. (D) Alignment of BlpC alleles found in *S. pseudopneumoniae* with known BlpC alleles of S. pneumoniae. (E) Alignment of CSP alleles found in *S. pseudopneumoniae*. Vertical lines indicate the cleavage site of the mature peptides from the leader sequence. Dashes represent gaps in the alignment. Download FIG S3, EPS file, 2.3 MB. Copyright © 2019 Garriss et al. 2019 Garriss et al. This content is distributed under the terms of the Creative Commons Attribution 4.0 International license.

10.1128/mBio.01286-19.9 FIG S4 Schematic representation of genetic elements encoding antibiotic resistance genes. (A) Mega-2 element carrying *mef*(E) and *msr*(D). (B) Integrating conjugative elements Tn*5251* and Tn*3872* (Tn*5251 + *Tn*917*), respectively, carrying *tet*(M) and *tet*(M)-*erm*(B). Arrows indicate ORFs pointing in the direction of transcription. Colors indicate the function of each ORF. ORFs flanking the different integration sites detected are indicated. The closest homolog in IS7493 is stated when possible. Download FIG S4, EPS file, 1.0 MB. Copyright © 2019 Garriss et al. 2019 Garriss et al. This content is distributed under the terms of the Creative Commons Attribution 4.0 International license.

Additionally, we investigated the presence of antibiotic resistance. Resistance to erythromycin and tetracycline were the most common among our LRTI isolates (Table 3). More than half of the *S. pseudopneumoniae* genomes (*n* = 24) harbored genes encoding resistance to tetracycline [*tet*(M)], 14- and 15-membered macrolides [*mef*(E) and *msr*(D)], and/or macrolides, lincosamides, and streptogramin B (MLS_B_ antibiotics) [*erm*(B)]. *mef*(E) and *msr*(D) were encoded by Mega-2 elements (macrolide efflux genetic assembly) integrated within the coding sequence of a DNA-3-methyladenine glycosylase homolog to SP_RS00900 of S. pneumoniae TIGR4 (Fig. S4A). Integration of Mega-2 in this site has been previously reported in S. pneumoniae (31, 32). Nine of the 11 strains carrying Mega-2 belonged to a subset of clade III, and the presence of this element was almost strictly associated with the absence of a plasmid (Fig. 7B). *tet*(M) and *erm*(B) genes were found within the Tn*916-*like integrating conjugative elements (ICEs) Tn*5251* (33) and Tn*3872* (34) (Fig. S4B). Tn*5251* and Tn*3872* ICEs were integrated in 7 different integration sites (Fig. 7B and Fig. S4C). Four of the integration sites were unique, while the other 3 were shared by two or more strains. ICE integration sites were mostly shared by closely related strains. One strain carried the *tet*(O) gene, which also encodes tetracycline resistance, and 2 other strains carried an aminoglycoside-3′-phosphotransferase [*aph*(3′)-Ia] gene.

**TABLE 3:** Antibiotic susceptibility profile of LRTI *S. pseudopneumoniae* isolates^*a*^

Strain	MIC (μg/ml) for:					
	SXT	Penicillin G	Erythromycin	Clindamycin	Tetracycline	Levofloxacin
BHN868	0.064	0.0004	2	0.5	32	0.5
BHN871	1.5	0.047	1.5	0.5	64	1
BHN877	4	0.064	4	0.5	32	1
BHN879	0.047	0.0004	0.19	0.125	0.5	0.5
BHN880	0.25	0.012	0.19	0.125	0.25	1
BHN881	2	0.125	6	0.5	48	0.75
BHN885	0.032	0.0008	3	0.094	32	1
BHN886	0.032	0.0008	4	0.094	0.094	0.5
BHN890	0.064	0.0008	0.25	0.125	0.25	1
BHN891	0.064	0.0008	0.125	0.094	32	0.75
BHN892	0.064	0.0008	0.125	0.064	0.25	0.5
BHN893	0.047	0.0008	0.125	0.125	0.5	0.75
BHN912	0.047	0.0008	0.125	0.094	0.5	1
BHN913	0.064	0.004	2	0.125	32	1.5
BHN914	0.064	0.016	0.125	0.125	0.5	0.75
BHN915	0.064	0.016	0.19	0.125	0.5	0.75
BHN916	0.094	0.0008	0.125	0.125	0.38	0.75
BHN918	0.064	0.016	0.25	0.125	0.5	0.75
BHN919	0.125	0.008	4	0.125	32	0.75
BHN920	0.064	0.006	0.125	0.125	0.38	1
BHN922	0.032	0.008	0.19	0.125	0.38	1
BHN1333	0.064	0.008	0.125	0.125	0.25	0.75
BHN1334	0.094	0.008	0.125	0.125	0.19	1.5
BHN1335	0.19	0.008	0.19	0.19	0.38	0.75
BHN1336	0.094	0.008	0.125	0.094	0.19	0.5
BHN1337	0.047	0.008	0.094	0.094	0.38	1.5
BHN1338	0.023	0.008	0.125	0.125	0.38	2
BHN1339	0.064	0.008	0.19	0.125	0.5	1
BHN1340	0.38	0.016	8	0.5	0.38	1.5
Nonsusceptible^*b*^	10.34 (3)	0 (0)	31.03 (9)	17.24 (5)	27.59 (8)	0 (0)

a MICs were interpreted using the CLSI guidelines for viridans streptococci (44), except for SXT, which was interpreted according to EUCAST guidelines (45) for non-meningitis S. pneumoniae isolates.

b Values are shown as % (*n*).

Phenotypic resistance to penicillin and co-trimoxazole (SXT) had high prevalence in other reports (4, 6–8) and were available for many of the NCBI genomes. We found that these types of resistance were also strongly associated with clade III. Taken together, 19/20 strains (95.2%) of clade III carried at least one genetic element encoding an AMR determinant or were shown to be resistant to at least one antibiotic (Fig. 7B). In contrast, a relatively small percentage of strains belonging to clade II (31.6%) were associated with AMR.

## DISCUSSION

Correct identification of SMG isolates remains a challenge to this day and impairs our understanding of their epidemiology and contribution to human disease. The high genetic relatedness of S. pneumoniae and *S. pseudopneumoniae*, exemplified by our result that they share 50% of their pangenomes, is likely due to their ability to acquire genetic material through natural transformation. Even though *S. pseudopneumoniae* causes milder infections than S. pneumoniae and is associated with underlying diseases (5, 8), it has been isolated from normally sterile body sites (5, 7). A causative agent has not been identified in a significant percentage of LRTI (≈40%) and community-acquired pneumonia cases, both in the community and hospital settings (35), and it is possible that a fraction of these cases are due to potential pathogens such as *S. pseudopneumoniae*, which might be discarded as commensals and for which reliable identification methods are lacking. Hence, some LRTI isolates included in this study could only be classified using WGS and phylogenetic analyses. By performing a thorough comparative genomic analysis, we identified for the first time a genetic marker that is entirely specific for *S. pseudopneumoniae*.

Our results show that pneumococcal genes known to be differentially regulated under infection- and colonization-relevant conditions are widespread in *S. pseudopneumoniae* and that only a surprisingly small percentage (5.6%) of them are absent from *S. pseudopneumoniae*. We further report the first *S. pseudopneumoniae* isolate encoding and expressing a capsule. The lack of transposase genes on either side of the capsule locus and its higher similarity to the capsular locus of an S. mitis strain suggest it was acquired from S. mitis rather than from a pneumococcal strain. Interestingly, a high prevalence of pneumococcal serotype 5 antigens in urine samples in the absence of culture confirmation has been reported in one study of community-acquired pneumonia cases in the United States (36). The possibility for *S. pseudopneumoniae* and other SMG species (37) to express the pneumococcal serotype 5 capsule should be taken into consideration when interpreting results based solely on serotype-specific assays. Further studies are needed to understand the role of the capsule in *S. pseudopneumoniae* and to evaluate the prevalence of encapsulated isolates in larger clinical collections.

The multiple pneumococcal virulence and colonization factors found in the core genome of *S. pseudopneumoniae* confirm earlier observations (3, 16). The presence of some crucial virulence factors, such as pneumolysin (Ply), could mark an important difference between *S. pseudopneumoniae* and the more commensal S. mitis, especially in light of our recent study in which Ply was shown to drive internalization of pneumococci within nonlysosomal compartments of immune cells commonly found in the lungs (38). In addition, the presence of large numbers of surface-exposed proteins could provide an advantage for adhesion and colonization, as was described for nonencapsulated pneumococci (17, 18). Surface-exposed proteins are important players for successful pneumococcal colonization, which constitutes the first step of pneumococcal disease, and display a wide variety of functions, from virulence to fitness and antibiotic tolerance (39, 40). In this scenario, the lack of a capsule might avoid restricting the ability of surface-exposed proteins to interact with their ligands on host cells (24). The large number of two-component signaling systems in *S. pseudopneumoniae* suggests that it is equipped to fine-tune its response to different environmental cues.

Our observations reveal a composite scenario of genetic elements in *S. pseudopneumoniae*, where prophages are abundant and plasmids and AMR-encoding ICEs are found in a large number of isolates. The fact that the core genome phylogeny delineates clades that harbor different genetic elements suggests that small differences in their core genome play a role in the maintenance or exclusion of these elements. Our findings suggest multiple acquisition events and subsequent clonal expansion of Tn*916*-like ICEs in *S. pseudopneumoniae* or intrachromosomal mobilization. Most of the strains carrying a Mega-2 element were found in a subset of the same clade, suggesting that its presence is mainly driven through clonal expansion, as was suggested for S. pneumoniae (31, 32). Besides genetic determinants of AMR, phenotypic resistance also showed a tight association with a specific lineage. In S. pneumoniae, longer durations of carriage are associated with increased prevalence of resistance (41). No specific known virulence factor except for PcpA could be specifically associated with a given *S. pseudopneumoniae* clade. However, the 4 PcpA^+^ strains as well as the 3 septicemia isolates belong to the same phylogenetic clade, which is also characterized by fewer AMR determinants. It will be interesting in the future to evaluate the relative virulence of strains belonging to different clades.

In conclusion, our single specific molecular marker for identifying *S. pseudopneumoniae* from other SMG species will be a useful resource for better understanding the clinical importance of this species. Moreover, our results reveal the impressive amount of surface-exposed proteins encoded by some strains and shed light on the overall distribution in *S. pseudopneumoniae* of genes known to be important during pneumococcal invasive disease and colonization.

## MATERIALS AND METHODS

### Bacterial isolates and molecular typing.

Thirty-two alpha-hemolytic strains isolated from sputum or nasopharyngeal swabs of patients with lower-respiratory-tract infections collected during the GRACE study (19) and presenting atypical results in traditional biochemical tests to identify S. pneumoniae were included in this study. Isolates were tested for optochin susceptibility and bile solubility (7, 42) by PCR for pneumococcal markers (*lytA*, *cpsA*, *spn_9802*, 16SrRNA) and by RFLP for pneumococcus-specific signatures (*lytA*, *ply-mly*) (7). BHN880 was serotyped by gel diffusion (43). MICs to all antibiotics were determined using Etests (bioMérieux) and interpreted using the Clinical and Laboratory Standards Institute (CLSI) guidelines for viridans streptococci (44), except for SXT, which was interpreted using the European Committee on Antimicrobial Susceptibility Testing (EUCAST) breakpoints for non-meningitis S. pneumoniae (45).

### Whole-genome sequencing, assembly, and phylogenetic analysis.

Chromosomal DNA was prepared from overnight cultures on blood agar plates using the genomic DNA buffer set and Genomic-tip 100/G (Qiagen) by following the manufacturer’s instructions. Long DNA insert sizes were used, and libraries were prepared with the Illumina TruSeq HT DNA sample preparation kit. Two-hundred-fifty-bp-long paired-end reads were generated. Adapters were removed from the demultiplexed reads, and reads were quality trimmed using Trimmomatic (46). The 24 genomes were assembled *de novo* with SPADES (v3.1.1) (47), annotated with PROKKA (v1.11) (48, 49), and deposited in NCBI (SOQB00000000 to SOQV00000000 [see Data Set S1 in the supplemental material]). Assembly metrics were calculated with QUAST 4.5.4 (49). kSNP 3.1 (50) was used to generate the SNP-based phylogenetic tree of SMG genomes. The optimum k-mer value of 19, estimated from Kchooser, and a consensus parsimony tree based on all the SNPs generated by kSNP were used (50). The phylogenetic tree was visualized in MEGA7 (51).

### Pangenome analysis, construction of SPPN species tree, and identification of virulence factors.

The pangenome analysis of orthologous gene clusters, species trees, and their respective gene trees were analyzed using panX (52) for the 39 completed S. pneumoniae genomes (pan:SPN), 44 *S. pseudopneumoniae* genomes (pan:SPPN), and both species (pan:SPPN-SPN) with default cutoff values. pan:SPPN analysis resulted in 885 core genes (strict core; 100% present in all strains), and the core genome tree/species tree for the SPPN species was constructed based on the core genome SNPs, including only single-copy core genes (*n* = 793). Using pan:SPPN-SPN, all COGs were queried for S. pneumoniae locus tags corresponding to 356 IPD/ECC genes (24) and 92 well-studied pneumococcal genes (21) listed in Data Set S2 and Table S3. Proteins listed in Table S3 were analyzed using a 70% length cutoff to score proteins as present; conservation of synteny was confirmed for all proteins. Genetic loci of proteins scored as absent were manually checked for contig breaks and pseudogenes.

### Molecular markers and PCR assay.

Thirty COGs unique to the 44 *S. pseudopneumoniae* genomes and absent from the 39 S. pneumoniae genomes (Table S1) were filtered from the pangenome analysis (pan:SPPN_SPN) and subjected to BLAST searches against all NCBI genomes, which included 8,358 S. pneumoniae genomes. The 44 nucleotide sequences of the two unique ORFs (SPPN_RS10375 and SPPN_RS06420) were aligned using the ClustalW algorithm in Geneious, version 10.1.3 (https://www.geneious.com), with default parameters (gap open cost, 15; gap extend cost, 6.66). The upstream (70 bp) and downstream (329 bp) intergenic regions of SPPN_RS10375 were included. Primers SPPN_RS10375F (5′-CTAATTGCTACTGCTATTTCCGGTG-3′) and SPPN_RS10375R (5′-CTGATACCTGCAACAAAAATCGAAG-3′) were designed in regions of 100% identity. PCR was performed using Phusion flash high-fidelity PCR master mix (ThermoFisher) by following the manufacturer’s instructions with an annealing temperature of 50°C. One μl of lysate, prepared by resuspending 2 to 3 isolated colonies in 100 μl Tris-EDTA containing 0.1% Triton and incubating at 98°C for 5 min, was used as the template. PCR products were run on a 1.2% agarose gel stained with GelRed (Biotium).

### *In silico* identification of new putative virulence features.

A database was built using the concatenated sequence of all proteins from the 44 *S. pseudopneumoniae* genomes and was queried for the conserved choline-binding domain COG5263 and the peptidase_M26 domain pfam07580/cl06563 to identify novel choline-binding proteins and ZMPs, respectively, using the NCBI Batch CD-Search tool (53). Novel two-component signal transduction systems (TCSs) were identified by finding proteins containing the HATPase domain of histidine kinase (cd00075/smart00387/pfam02518) that were immediately preceded or followed by a DNA-binding regulator possessing the signal-receiver domain cd00156.

### Analysis of capsular loci.

Homologues of *cpsA* and *wzg* were searched for in pan:SPPN_SPN using gene family SP_RS01690. The locus was subsequently checked manually for the presence of the complete locus [BHN880_01411 to BHN880_01431]. The retrieved *cps* locus was subjected to a BLASTN search to identify the closest homologs. Pairwise alignment with the S. pneumoniae Ambrose serotype 5 locus (CR931637.1) (54) and S. mitis 21/39 (AYRR01000010.1) *cps* locus was performed using Easyfig (55).

### Hemolysis assay.

Bacteria were grown overnight on blood agar plates at 37°C in 5% CO_2_. S. pneumoniae strains were grown into C+Y medium until exponential phase (optical density at 620 nm [OD_620_] of 0.4). *S. pseudopneumoniae* strains were grown in C+Y medium to an OD_620_ of 0.3 and then inoculated into a secondary culture, which was grown to an OD_620_ of 0.25. Dilutions were made to obtain the desired concentration of bacterial cells, and viable counts were performed to retrospectively confirm bacterial numbers. Blood from healthy human donors (obtained from Karolinska University Hospital) was diluted 1:100 in phosphate-buffered saline–0.5 mM dithiothreitol, mixed 1:1 with 2-fold serial dilutions of S. pneumoniae or *S. pseudopneumoniae* cultures in 96-well plates, and incubated at 37°C for 1 h. After 50 min of incubation, 0.1% Triton X-100 was added to the positive-control wells. Cells were spun down at 400 × *g* for 15 min, and the absorbance of the supernatants was measured at 540 nm in a microplate reader. Percentage of lysis compared to the positive control was calculated. All strains were tested in triplicate.

### *In silico* identification of AMR determinants, plasmids, and phages.

The 44 genomes were screened in Resfinder 3.0 (56) for the acquired AMR genes (90% identity threshold, minimum length of 60%). Chromosomal genes flanking Tn*916-*like ICEs were defined by using BLASTn to retrieve the loci in strain IS7493 (NC_015875.1) of the genes located immediately upstream of the integrase and immediately downstream of *orf24* of Tn*5251* (FJ711160.1). Genome assemblies were queried for genes associated with known S. pneumoniae and S. mitis phages (SPH_0026, IPP61_00001, SPH_0070, SP670_2134, SP670_0091, SM1p01, SPPN_RS05280, and HMPREF1112_1362) and the *S. pseudopneumoniae* plasmid pDRPIS7493 (NC_015876.1). Phage sequences were manually analyzed and deemed full length if they started with an integrase gene, ended with a lytic amidase, and were ≥30 kb in length.

### Data availability.

All 21 *S. pseudopneumoniae* sequenced genomes have been deposited in GenBank under BioProject code PRJNA528011, BioSample numbers SAMN11166137 to SAMN11166157, and accession numbers SOQV00000000, SOQU00000000, SOQT00000000, SOQS00000000, SOQR00000000, SOQQ00000000, SOQP00000000, SOQO00000000, SOQN00000000, SOQM00000000, SOQL00000000, SOQK00000000, SOQJ00000000, SOQI00000000, SOQH00000000, SOQG00000000, SOQF00000000, SOQE00000000, SOQD00000000, SOQC00000000, and SOQB00000000. The accession numbers of the other genomes used in this study are listed in Data Set S1.
