# Supplementary material for: Genomic Characterization of the Emerging Pathogen Streptococcus pseudopneumoniae
Source: mBio. 2019 Jun 25;10(3):e01286-19. doi: 10.1128/mBio.01286-19 (PMC6593409; doi:10.1128/mBio.01286-19)
Supplement: TABLE S1 [file mBio.01286-19-st001.pdf]

Table S1 ***S. pseudopneumoniae*** **molecular marker candidates**

IS7493 Locus tag	Annotation	CDS length (bp)	BLAST hits in other species[a]
SPPN_RS00255	hypothetical protein	313	*Mycobacterium tuberculosis*
SPPN_RS00500	Cbp2	898	*S. pneumoniae*
SPPN_RS00555	Cbp4	1188	*S.pneumoniae, S. mitis*
SPPN_RS00560	C/BlpC family peptide pheromone/bacteriocin assocated with T(	147	*S. mitis*
SPPN_RS00565	HK14	1290	*S. mitis*
SPPN_RS00635	ethanolamine utilization protein EutH	151	*S. mitis*
SPPN_RS00640	class IIb bacteriocin	194	*S. mitis, S. oralis*
SPPN_RS02030	thiol-activated cytolysin	1912	*S.pneumoniae, S. mitis*
SPPN_RS02225	CbpF	993	*S. oralis*
SPPN_RS02970	toxin Fic	996	*Streptococcus sp.* M334, *S. oralis, S. infantis, S. mitis*
SPPN_RS03570	RR16	726	*S. canis*
SPPN_RS04580	twin-arginine translocase subunit TatC	732	*S. mitis*
SPPN_RS04585	twin-arginine translocase TatA/TatE family subunit	171	*S. mitis*
SPPN_RS04595	RelB antitoxin	328	*S. mitis*
SPPN_RS04720	hypothetical protein	440	*S. mitis*
**SPPN_RS06420**	**aminotransferase class V-fold PLP-dependent enzyme**	**352**	**-**
SPPN_RS06710	LTA synthase family protein	2151	*S. mitis*
SPPN_RS07710	molybdopterin biosynthesis protein MoeB	1134	*Streptococcus sp.* M334
SPPN_RS07720	ABC transporter permease	718	*Chlamydia*
SPPN_RS08060	type II toxin-antitoxin system RelE/ParE family toxin	351	*S.pneumoniae, S. mitis*
SPPN_RS08065	XRE family transcriptional regulator	288	*S. mitis*
SPPN_RS08575	ArsR family transcriptional regulator	287	*S. mitis*
SPPN_RS08580	CPBP family intramembrane metalloprotease	900	*S. mitis*
SPPN_RS08950	LrgB family protein	696	*S. mitis*
SPPN_RS09290	7-cyano-7-deazaguanine synthase	654	*S.pneumoniae, S. mitis*
SPPN_RS09295	6-carboxytetrahydropterin synthase QueD	444	*S. mitis*
SPPN_RS09630	ABC transporter ATP-binding protein	1737	*S. mitis*
SPPN_RS10230	XRE family transcriptional regulator	919	*S. mitis, S. oralis*
**SPPN_RS10375**	**hypothetical protein**	**240**	**-**
SPPN_RS10785	Toxic anion resistance protein (TelA)	1248	*S. mitis*

[a] Species in which BLASTp hits were found. The presence of each candidate was also tested by BLASTn against the 8,358 S. pneumoniae NCBI genomes.
