# Supplementary figures and images for: Genomic Characterization of the Emerging Pathogen Streptococcus pseudopneumoniae
Source: mBio. 2019 Jun 25;10(3):e01286-19. doi: 10.1128/mBio.01286-19 (PMC6593409; doi:10.1128/mBio.01286-19)

Figure S1

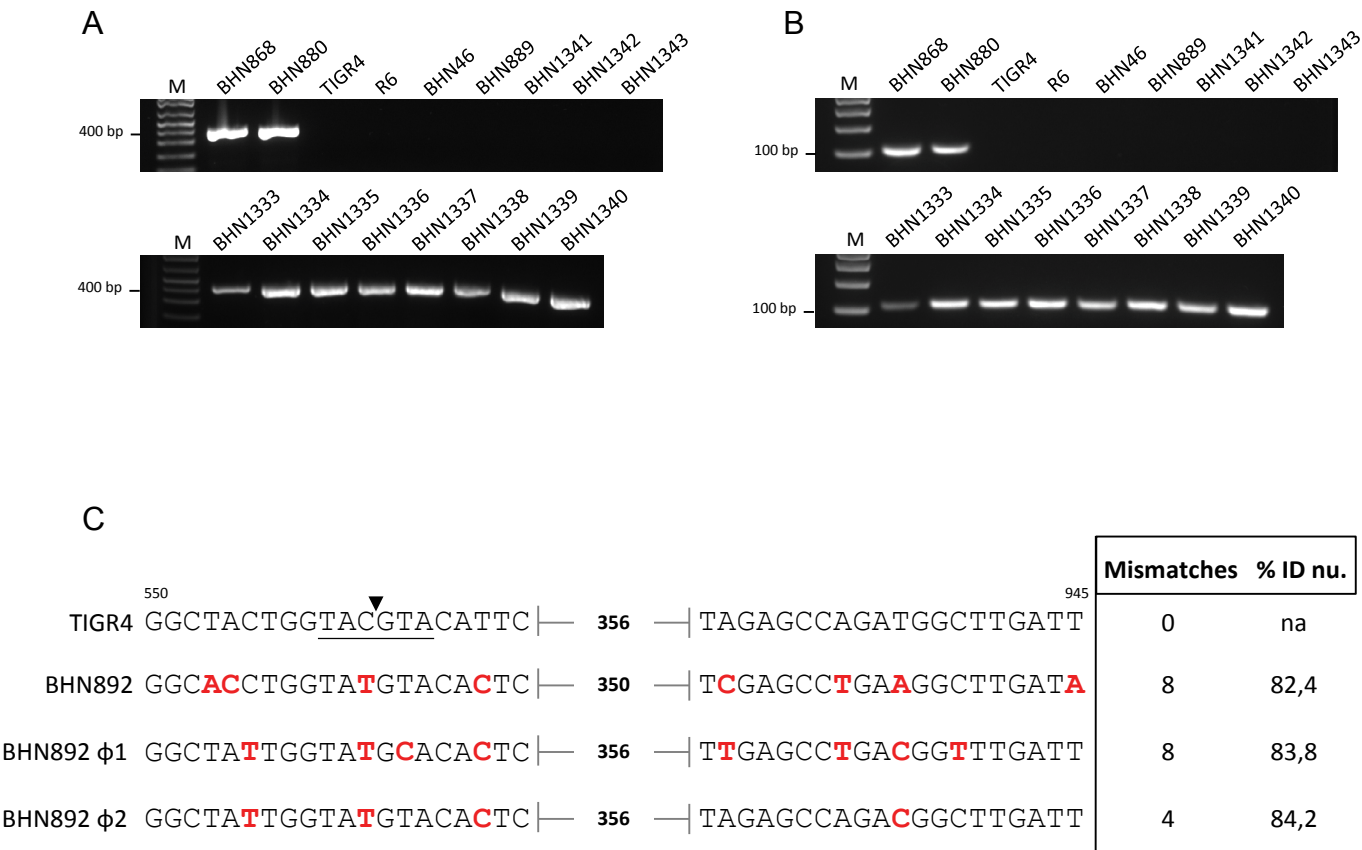

Supplement: FIG S1 [file mBio.01286-19-sf001.pdf]
