# Supplementary material for: Genomic Characterization of the Emerging Pathogen Streptococcus pseudopneumoniae
Source: mBio. 2019 Jun 25;10(3):e01286-19. doi: 10.1128/mBio.01286-19 (PMC6593409; doi:10.1128/mBio.01286-19)
Supplement: TABLE S3 [file mBio.01286-19-st003.pdf]

Table S3 Locus tags of *S. pneumoniae* used for the analysis of virulence and colonization genes in *S. pseudopneumoniae*

		Locus tag *Spn* [a]	Locus tag *Sppn* IS7493[b]
LPxTG - Proteins	PrtA	SP_RS03145	SPPN_RS03300
	MucB	SP_RS07345	SPPN_RS07195
	ZmpB	SP_RS03260	SPPN_RS03420
	EndoD	SP_RS02450	SPPN_RS02880
	StrH	SP_RS00325	SPPN_RS00780
	PavB	SP_RS00425	SPPN_RS00855
	ZmpA	SP_RS05695	SPPN_RS12030
	BgaA	SP_RS03175	SPPN_RS03340
	PfbA	SP_RS09095	E3V19_04350
	SpuA	SP_RS01315	SPPN_RS02025
	ZmpD	SPJ_1074	E3V19_11415
	Eng	SP_RS01795	E3V19_01805
	ZmpC	SP_RS00380	NA
	HysA	SP_RS01530	NA
	PclA	spr1403	NA
	PsrP	SP_RS12370	NA
	KsgA	spr1806/SP_1992/SP_RS10010	NA
	RrgA	SP_RS02280	NA
	RrgB	SP_RS02285	NA
	RrgC	SP_RS02290	NA
	PitA	SPT_RS05230	NA
	PitB	SPT_RS05240	NA
	NanA	spr1536	SPPN_RS08175
Choline-Binding Proteins (CBPs)	LytA	SP_RS09740	SPPN_RS09700
	LytB	SP_RS04785	SPPN_RS07065
	LytC	SP_RS07755	SPPN_RS07615
	CbpE	SP_RS04600	SPPN_RS06520
	CbpD	SP_RS11240	SPPN_RS11125
	CbpF	SP_RS01930	SPPN_RS02225
	CbpC	SP_RS01845	SPPN_RS02185
	CbpJ	SP_RS01850	SPPN_RS02190
	CbpG	SP_RS01925	SPPN_RS02230
	CbpL	SP_RS03275	SPPN_RS03435
	PcpA	SP_RS10900	SPPN_RS02450
	PspC	SP_RS11185	E3V19_07630
	CbpI	SP_RS00375	NA
	PspA	SP_RS00595	NA
Lipoproteins	PsaA	SP_RS08145	SPPN_RS07960
	PhtD	SP_RS04975	SPPN_RS05005
	AliA	SP_RS01790	SPPN_RS02130
	PiuA	SP_RS09285	SPPN_RS09420
	PpiA	SP_RS03765	SPPN_RS03845
	PpmA	SP_RS04865	SPPN_RS04905
	PhtE	SP_RS04980	E3V19_09845
	GlnQ	SP_RS02995	E3V85_00790
	PiaA	SP_RS05120	AE262_RS05960
	PhtA	SP_RS05790	NA
	PhtB	SP_RS05785	NA
Non-classical surface exposed Proteins	Eno	SP_RS05590	SPPN_RS05535
	GAPDH	SP_RS10185	SPPN_RS10215
	HtrA	SP_RS11450	SPPN_RS11310
	PavA	SP_RS04790	SPPN_RS07060
	Pbp1B	SP_RS10690	SPPN_RS10515
	6PGD	SP_RS01835	SPPN_RS02170
	StkP	SP_RS08570	SPPN_RS09025
	Usp45	SP_RS11315	SPPN_RS11185
	Ply	SP_RS09670	SPPN_RS09655
	PppA	SP_RS07750	SPPN_RS07610
	Fic-Like	SP_RS02805	SPPN_RS02990
	NanB	SP_RS08335	SPPN_RS08130
	NanC	SP_RS06510	E3V14_08425
	HK01	SP_RS08045	SPPN_RS07855
	RR01	SP_RS08050	SPPN_RS07860

**Two component signalling systems**	**HK02**	SP_RS06010	SPPN_RS05865
	**RR02**	SP_RS06015	SPPN_RS05870
	**HK03**	SP_RS01895	SPPN_RS02250
	**RR03**	SP_RS01900	SPPN_RS02245
	**HK04**	SP_RS10605	SPPN_RS10440
	**RR04**	SP_RS10600	SPPN_RS10435
	**HK05**	SP_RS03910	SPPN_RS04035
	**RR05**	SP_RS03905	SPPN_RS04030
	**HK06**	SP_RS11195	E3V19_07640
	**RR06**	SP_RS11200	E3V19_07645
	**HK07**	SP_RS00800	SPPN_RS01475
	**RR07**	SP_RS00805	SPPN_RS01480
	**HK08**	SP_RS00435	SPPN_RS00865
	**RR08**	SP_RS00430	SPPN_RS00860
	**HK09**	SP_RS03250	SPPN_RS03410
	**RR09**	SP_RS03255	SPPN_RS03405
	**HK10**	SP_RS02970	SPPN_RS03140
	**RR10**	SP_RS02965	SPPN_RS03135
	**HK11**	SP_RS10060	SPPN_RS10155
	**RR11**	SP_RS10055	SPPN_RS10150
	**HK12**	SP_RS11430	SPPN_RS11290
	**RR12**	SP_RS11425	SPPN_RS11285
	**HK13**	SP_RS02580	SPPN_RS08735
	**RR13**	SP_RS02575	SPPN_RS08740
	**ORR**	SP_RS01840	SPPN_RS02175
**Stand-alone regulators**	**PsaR**	SP_RS08075	SPPN_RS07880
	**MerR**	SP_RS03620	SPPN_RS03700
	**RlrA**	SP_RS02275	NA
	**MgrA**	SP_RS08935	NA
**Novel Choline-Binding Proteins (CBPs)**	**Cbp1**		E3V42_08495
	**Cbp2**		SPPN_RS00500
	**Cbp3**		SPPN_RS00550
	**Cbp4**		SPPN_RS00555
	**Cbp5**		SPPN_RS00615
	**Cbp6**		SPPN_RS00620
	**Cbp7**		SPPN_RS00890
	**Cbp8**		SPPN_RS00895
	**Cbp9**		SPPN_RS01860
	**Cbp10**		SPPN_RS02560
	**Cbp11**		SPPN_RS02565
	**Cbp12**		SPPN_RS08165
	**Cbp13**		SPPN_RS09910
	**Cbp14**		SPPN_RS09915
	**Cbp15**		E3V19_02560
	**Cbp16**		E3V19_02565
	**Cbp17**		E3V19_11840
	**Cbp18**		E3V19_02690
	**Cbp19**		E3V91_01480
**Novel Two-component systems**	**HK14**		SPPN_RS00565
	**RR14**		SPPN_RS00570
	**HK15**		SPPN_RS01890
	**RR15**		SPPN_RS11635
	**HK16**		SPPN_RS03565
	**RR16**		SPPN_RS03570
	**HK17**		SPPN_RS07700
	**RR17**		SPPN_RS07705
	**HK18**		E3V59_10385
	**RR18**		E3V59_10390
	**HK19**		E3V34_05535
	**RR19**		E3V34_05540
**Novel ZMPs**	**ZmpE**		SPPN_RS10975
	**ZmpF**		E3V19_09485

[a] *S. pneumoniae* (*Spn*) locus tag used for the analysis.

[b] Locus tag in *S. pseudopneumoniae* IS7493 is indicated when present.
When absent from IS7493 the locus tag in another strain is indicated.
