# Supplementary material for: Genomic Characterization of the Emerging Pathogen Streptococcus pseudopneumoniae
Source: mBio. 2019 Jun 25;10(3):e01286-19. doi: 10.1128/mBio.01286-19 (PMC6593409; doi:10.1128/mBio.01286-19)
Supplement: TABLE S2 [file mBio.01286-19-st002.pdf]

Table S2 Prophages

Strain	Integrase[a]	Upstream gene[b]	Dowsntream gene[c]	Phage completedness[d]
1321	*intSppn1*	SPPN_RS05275	SPPN_RS05395	B
5247	*intSppn1*	SPPN_RS05275	SPPN_RS05395	A
5305	*intSppn1*	SPPN_RS05275	SPPN_RS05395	A
22725	*int2a*	SPPN_RS11435	NA	C
	intSppn1	SPPN_RS05275	SPPN_RS05395	C
276-03	*int2a*	SPPN_RS08475	SPPN_RS08470	C
	intSppn1	SPPN_RS05275	SPPN_RS05395	C
338-14	*int2a*	SPPN_RS07570	SPPN_RS07555	C
	intSppn1	SPPN_RS05275	SPPN_RS05395	C
61-14	*int2a*	SPPN_RS07570	SPPN_RS07555	C
	intSppn1	SPPN_RS05275	SPPN_RS05395	C
ATCC_BAA-960	*int2a*	SPPN_RS07570	SPPN_RS07555	C
	intSppn1	NA	NA	C
BHN868	*int2a*	SPPN_RS07570	SPPN_RS07555	C
	intSppn1	SPPN_RS05275	SPPN_RS05395	C
BHN871	*int2a*	SPPN_RS07570	SPPN_RS07555	B
	intSppn1	SPPN_RS05275	SPPN_RS05395	A
BHN877	*int2a*	SPPN_RS07570	SPPN_RS07555	C
	intSppn1	SPPN_RS05275	SPPN_RS05395	B
BHN879	*int2a*	SPPN_RS07570	SPPN_RS07555	C
	intSppn1	SPPN_RS05275	SPPN_RS05395	C
BHN880	*int2a*	SPPN_RS07570	SPPN_RS07555	C
	intSppn1	SPPN_RS05275	SPPN_RS05395	C
BHN881	*int2a*	SPPN_RS07570	SPPN_RS07555	B
	intSppn1	SPPN_RS05275	SPPN_RS05395	A
BHN885	*int2a*	SPPN_RS07570	SPPN_RS07555	B
	intSppn1	SPPN_RS05275	SPPN_RS05395	B
BHN886	*int2a*	SPPN_RS07570	SPPN_RS07555	C
	intSppn1	SPPN_RS05275	SPPN_RS05395	C
BHN890	*int2a*	SPPN_RS07570	SPPN_RS07555	B
	intSppn1	SPPN_RS05275	SPPN_RS05395	A
BHN891	*int2a*	SPPN_RS07570	SPPN_RS07555	C
	intSppn1	SPPN_RS05275	SPPN_RS05395	C
BHN892	*int2a*	SPPN_RS07570	SPPN_RS07555	B
	intSppn1	SPPN_RS05275	SPPN_RS05395	B
BHN893	*int2a*	SPPN_RS07570	SPPN_RS07555	C
	intSppn1	SPPN_RS05275	SPPN_RS05395	C
BHN912	*int2a*	SPPN_RS07570	SPPN_RS07555	C
	intSppn1	SPPN_RS05275	SPPN_RS05395	C
BHN913	*int2a*	SPPN_RS07570	SPPN_RS07555	C
	intSppn1	SPPN_RS05275	NA	C
BHN914	*intSppn1*	SPPN_RS05275	SPPN_RS05395	A
BHN915	*int2a*	SPPN_RS07570	SPPN_RS07555	B
	intSppn1	SPPN_RS05275	SPPN_RS05395	A
BHN916	*int2a*	SPPN_RS07570	SPPN_RS07555	C
	intSppn1	SPPN_RS05275	SPPN_RS05395	C
BHN918	*int2a*	SPPN_RS07570	SPPN_RS07555	C
	intSppn1	SPPN_RS05275	SPPN_RS05395	C
BHN919	*intSppn1*	SPPN_RS05275	SPPN_RS05395	A
BHN920	*intSppn1*	SPPN_RS05275	SPPN_RS05395	A
BHN922	*int2a*	SPPN_RS07570	SPPN_RS07555	C
	intSppn1	SPPN_RS05275	SPPN_RS05395	C
CCUG 49455	*int2a*	SPPN_RS07570	SPPN_RS07555	C
	intSppn1	SPPN_RS05275	SPPN_RS05395	C
CCUG 62647	*intSppn1*	SPPN_RS05275	SPPN_RS05395	A
CCUG 63747	*intSppn1*	SPPN_RS05275	SPPN_RS05395	A
CipR71	*intSppn1*	NA	NA	A
G42	*int2a*	SPPN_RS07570	SPPN_RS07555	C
	intSppn1	NA	NA	C
IS7493	*int2a*	SPPN_RS11435	NA	C
	intSppn1	SPPN_RS05275	SPPN_RS05395	C
SK674	*intSppn1*	NA	NA	A
SMRU22	*intSppn1*	SPPN_RS05275	SPPN_RS05395	A
SMRU2248	*intSppn1*	SPPN_RS05275	SPPN_RS05395	C
SMRU2944	*intSppn1*	SPPN_RS05275	SPPN_RS05395	A
SMRU688	*intSppn1*	SPPN_RS05275	SPPN_RS05395	A
SMRU689	*int2a*	SPPN_RS07570	SPPN_RS07555	C
	intSppn1	SPPN_RS05275	NA	C
SMRU737	*int2a*	SPPN_04175	NA	C
	intSppn1	SPPN_RS05275	SPPN_RS05395	C
SMRU856	*intSppn1*	SPPN_RS05275	SPPN_RS05395	A
SMRU90	*int2a*	SPPN_RS07570	SPPN_RS07555	C
	intSppn1	SPPN_RS05275	SPPN_RS05395	C

[a] *intSppn1* , SPPN_RS05280; *int2a* , SPPN_RS08930

[b] Homologue in IS7493 of ORF immediately upstream the phage integrase. NA indicates the integrase was the first ORF in the contig.

[c] Homologue in IS7493 of the gene immediately downstream of the last phage gene. NA indicates the last prophage gene was the last in the contig.

[d] A, phage complete; B, assembly gap (1 or 2) but phage appears complete and large part of phage sequence in the same contig as the integrase;
C, impossible to assess if complete due to too many assembly gaps , or phage remnant
